# Interference of TRPV1 function altered the susceptibility of PTZ-induced seizures

**DOI:** 10.3389/fncel.2015.00020

**Published:** 2015-02-10

**Authors:** Yun-Fang Jia, Ying-Chao Li, Yan-Ping Tang, Jun Cao, Li-Ping Wang, Yue-Xiong Yang, Lin Xu, Rong-Rong Mao

**Affiliations:** ^1^Key Laboratory of Animal Models and Human Disease Mechanisms of Chinese Academy of Sciences & Yunnan Province, and KIZ/CUHK Joint Laboratory of Bioresources and Molecular Research in Common Disease, and Laboratory of Learning and Memory, Kunming Institute of Zoology, Chinese Academy of SciencesYunnan, China; ^2^Kunming College of Life Science, University of Chinese Academy of SciencesBeijing, China; ^3^School of Life Sciences, University of Science and Technology of ChinaHefei, China

**Keywords:** TRPV1, capsaicin, capsazepine, seizure, hippocampus, pentylenetetrazol

## Abstract

Transient receptor potential vanilloid 1 (TRPV1) is widely distributed in the central nervous system (CNS) including hippocampus, and regulates the balance of excitation and inhibition in CNS, which imply its important role in epilepsy. We used both pharmacological manipulations and transgenic mice to disturb the function of TRPV1 and then studied the effects of these alterations on the susceptibility of pentylenetetrazol (PTZ)-induced seizures. Our results showed that systemic administration of TRPV1 agonist capsaicin (CAP, 40 mg/kg) directly induced tonic-clonic seizures (TCS) without PTZ induction. The severity of seizure was increased in lower doses of CAP groups (5 and 10 mg/kg), although the latency to TCS was delayed. On the other hand, systemic administration of TRPV1 antagonist capsazepine (CPZ, 0.05 and 0.5 mg/kg) and TRPV1 knockout mice exhibited delayed latency to TCS and reduced mortality. Furthermore, hippocampal administration of CPZ (10 and 33 nmol/μL/side) was firstly reported to increase the latency to TCS, decrease the maximal grade of seizure and mortality. It is worth noting that decreased susceptibility of PTZ-induced seizures was observed in hippocampal TRPV1 overexpression mice and hippocampal CAP administration (33 nmol/μL/side), which is opposite from results of systemic agonist CAP. Our findings suggest that the systemic administration of TRPV1 antagonist may be a novel therapeutic target for epilepsy, and alteration of hippocampal TRPV1 function exerts a critical role in seizure susceptibility.

## Introduction

Epilepsy is characterized by the imbalance between excitation and inhibition of central nervous system (CNS) (Engel, 1996; Stafstrom, 2003), affecting 1–3% of the worldwide population (Bhalla et al., 2012). Current prescribed anti-epileptic drugs (AED) are not effective in all individuals with epilepsy (Fu et al., 2009; Hu et al., 2012), thus it is necessary to find novel therapeutic targets for epilepsy. Previous studies have showed that ion channels play pivotal roles in epilepsy, and most of them have focused on the selective ion channels such as sodium and potassium channels (Armijo et al., 2005; Lerche et al., 2013). However, little attention has been paid to the role of non-selective ion channels in epilepsy, which may have fewer side effects than the selective ion channels.

Transient receptor potential vanilloid 1 (TRPV1), the subfamily vanilloid member 1 (Ramsey et al., 2006), is a ligand-gated non-selective cation channel with high calcium permeability (Pedersen et al., 2005). It is well-known that TRPV1 is widely expressed in both rodents and human CNS including hippocampus (Mezey et al., 2000; Cristino et al., 2006; Menigoz and Boudes, 2011), which is often the focus of epileptic seizures (Passouant and Cadilhac, 1960; Stafstrom, 2003). Activation of TRPV1 was found to inhibit calcium influx and reduce GABA release in synaptosomal hippocampus preparations, thus increase the excitability of innervated pyramidal cells (Kofalvi et al., 2006; Gibson et al., 2008). In contrast, activation of presynaptic TRPV1 inhibited CA1 pyramidal neurons via increasing GABA output (Al-Hayani et al., 2001; Kofalvi et al., 2006). Taken together, these results imply that hippocampal TRPV1 may play an important role in epilepsy (Fu et al., 2009). However, the function of hippocampal TRPV1 in epileptogenesis *in vivo* has not been reported systematically.

It has been reported that agonist of TRPV1 enhanced but antagonist suppressed epileptiform activity (Gonzalez-Reyes et al., 2013) and hyper-excitability in hippocampus (Chen et al., 2007; Bhaskaran and Smith, 2010). In addition, TRPV1 agonist capsaicin (CAP) exhibited pro-convulsant activity, which could be blocked by the antagonist capsazepine (CPZ), and CPZ was able to prevent PTZ-induced seizures (Manna and Umathe, 2012). A recent study has reported that the severity of PTZ-induced seizures was decreased in TRPV1 deficiency mice (Kong et al., 2014). Furthermore, significantly increased expression of TRPV1 was found in the hippocampus and cortex of rodents and patients with temporal lobe epilepsy (Bhaskaran and Smith, 2010; Sun et al., 2013). The above studies suggest that TRPV1 activation may accelerate epileptogenesis, while the antagonists of TRPV1 may be the potential AED (Fu et al., 2009). Inconsistent with the antagonist results, piperine as an agonist of TRPV1 was reported to exert anti-seizure effect (Chen et al., 2013; Khom et al., 2013). Lee et al. also has found that CAP prevents kainic acid-induced epileptogenesis in mice (Lee et al., 2011). Thus, a systematic investigation is necessary to elucidate the effects of agonist and antagonist of TRPV1 on epileptogenesis and to clarify the opposite effects of CAP on epileptogenesis in previous reports.

Therefore, we studied the systemic effects of the TRPV1 agonist and antagonist on PTZ-induced seizures in a dose dependent manner. And we further evaluated PTZ-induced seizures in transgenic mice including TRPV1 knockout and hippocampal TRPV1 overexpression mice. Then, to clarify the function of hippocampal TRPV1 on epilepsy, the agonist and antagonist of TRPV1 were administrated into hippocampus respectively to detect the effects on PTZ-induced seizures. To the best of our knowledge, this is the first study to investigate the role of hippocampal TRPV1 in epileptogenesis *in vivo* using pharmacological strategies combined with genetic modified mice.

## Materials and methods

### Animals

All procedures and animal care were approved by the Animal Research Committees of the Kunming Institute of Zoology, Chinese Academy of Sciences, China. Animals were group-housed (4–5 animals per cage) in clear plastic cages under standard laboratory conditions that included a thermo-regulated temperature (22–24°C), 50% humidity, and a 12-h dark/ light cycle with free access to water and food. All behavioral experiments were performed between 10:00 and 17:00.

Adult male Kunming mice weighing 25–30 g and Sprague-Dawley (SD) rats weighing 250–300 g were used (Animal House Center, Kunming Medical College, Yunnan, China). TRPV1 knockout mice (Caterina et al., 2000) were obtained from the Model Animal Research Center (Nanjing University, Jiangsu, China), and matings between TRPV1 heterozygous mice produced offspring with expected Mendelian distributions of gender and genotype. Hippocampal TRPV1 overexpression mice were generated by crossing *ROSA-stop flox-TRPV1-IRES-ECFP* (hereafter called ROSA-TRPV1) mice (Arenkiel and Klein, 2008) to heterozygous *Cre* transgenic mice, T29-1, which are able to mediate *Cre/loxP* recombination exclusively in the hippocampal CA1 pyramidal cells (Tsien et al., 1996). ROSA-TRPV1 mice were obtained from the Jackson Laboratory (the stock number: 008513), and the *Cre* transgenic mice (hereafter called CA1-Cre) were obtained from the Model Animal Research Center (Nanjing University, Jiangsu, China). All mice were weaned on postnatal day 21 (P21).

### Drugs

Pentylenetetrazol (PTZ), ethosuximide (ESM), capsaicin (CAP), and capsazepine (CPZ) (Sigma Aldrich, St. Louis, USA) were freshly prepared prior to use. PTZ and ESM were dissolved in saline. CAP and CPZ were dissolved in a 1:1:8 mixture of Tween 80: ethanol: saline.

For the control (vehicle) group, animals received vehicle (1 mL/kg, i.p.), which is a 1:1:8 mixture of Tween 80: ethanol: saline. For the positive control group, animals received ESM (625 mg/kg, i.p.). For the test groups, mice were respectively injected with CAP (5, 10, and 40 mg/kg, i.p.) and CPZ (0.05, 0.5, and 1 mg/kg, i.p.). Animals received intra-hippocampal (i.h.) injections of 1 μL per side of ESM (200 nmol/μL/side), CAP (10, 33, and 100 nmol/μL/side), and CPZ (10, 33, and 100 nmol/μL/side) respectively. Thirty minutes later all animals were injected with PTZ (60 mg/kg, s.c.).

### PTZ-induced seizures

Animals were placed individually in Plexiglas boxes and seizure behaviors were observed for 30 min after PTZ injection (60 mg/kg, s.c.). The seizure intensity was evaluated as follows (Racine, 1972; Hansen et al., 2012): Stage 0, no response; Stage 1, ear and facial twitching; Stage 2, myoclonic jerks, convulsive waves through the body; Stage 3, clonic convulsion with forelimb clonus and rearing; Stage 4, clonic seizure with rolling over into a side position side position, loss of postural control; Stage 5, generalized tonic-clonic seizures (TCS), tonic extension episode and status epilepticus; and Stage 6, death within 30 min. Latency to the onset of TCS, the maximal seizure score (obtained during 30 min for rats; obtained in 5-min blocks during 30 min for mice), and the number of mortality were measured. The maximal latency was taken as 1800 s if no signs of seizures were observed during 30 min.

### Cannula implantation

Rats were anesthetized with pentobarbital sodium (80 mg/kg, i.p.). A guide cannula (22 gauge) was stereotaxically implanted in the CA1 region of hippocampus at coordinates AP-3.8, Lat. ± 2.8, DV 2.5 of the atlas of Paxinos and Watson (1986). The guide cannula were fixed with dental cement for which three small skull screws (1 mm) were previously screwed into the skull as anchors. Animals were then allowed to recover for one week. Drugs were administered with a microinjector connected to an internal cannula by polyethylene tubing, and a volume of 1 μL per side was administered over a period of 8 min. The volume of 1 μL per side injected into hippocampus is usually used in previous studies (Li et al., 2008; Mitsushima et al., 2013). The cannula was left in the place for a further 2 min before being slowly withdrawn to avoid back flow.

### The confirmation of injected location in hippocampus with trypanblue staining

After finishing all the experiments, a volume of 1 μL per side trypanblue was administered in the implanted rat hippocampus as the methods mentioned above. Then the rats were deeply and fast anesthetized by diethyl ether, and their brains were dissected in cold 0.01 M phosphate buffered saline (PBS; pH 7.4). Four hundred-μm brain sections were cut, and the images were captured using a stereoscopic microscope (Leica).

### Genotyping assay

Genomic DNA was extracted by the TIAN quick Midi Purification kit (Tiangen Biotech (Beijing) CO., LTD. DP 204-0F3). The targeted lines of DNA from the specific transgenic mice were detected by polymerase chain reaction (PCR). The specific primers sequences of the DNA from TRPV1 knockout mice, CA1-Cre mice and ROSA-TRPV1 mice were shown as follows:

TRPV1-Wild type Forward: CCTGCTCAACATGCTCATTG

TRPV1-Common: TCCTCATGCACTTCAGGAAA

TRPV1-Mutant Forward: CACGAGACTAGTGAGACGTG

Product length = 984 bp (wild type) or 600 bp (mutant)

Cre-Forward: TCGATGCAACGAGTGATGAG

Cre-Reverse: TCCATGAGTGAACGAACCTG

Product length = about 300–400 bp

ROSA-TRPV1-Wild type Forward: TCCCAAAGTCGCTCTGAGTT

ROSA-TRPV1-Common: ACTCGGGTGAGCATGTCTTT

ROSA-TRPV1-Mutant Forward: GCATGGACGAGCTGTACAAG

Product length = 486 bp (wild type) or 600 bp (mutant)

The information of TRPV1 and ROSA-TRPV1 primers were obtained from Jackson Laboratory.

### Reverse transcription PCR (RT-PCR) assay

The hippocampal tissues of TRPV1 knock out mice and hippocampal TRPV1 overexpression mice were immediately isolated on ice after diethyl ether anesthesia. Thirty minutes after PTZ-induction (60 mg/kg, s.c.), the hippocampal tissues of the mice were immediately isolated on ice after diethyl ether anesthesia. Total RNA was extracted from hippocampal tissues of mice using RNA simple Total RNA kit (Tiangen Biotech (Beijing) CO., LTD. DP 419). And then the cDNA was synthesized by the Prime Script™ Reverse Transcriptase Reagent kit (Takara, RR037A), which was performed according to the manufacturer's instructions. The relative mRNA expression level of TRPV1 was measured by RT-PCR. The background density was subtracted from the TRPV1 band density and normalized to β-actin, which was used as the loading control gene. The specific primers sequences of TRPV1 and β-actin were displayed as follows:

TRPV1-Forward: GGGTCATTTCTCCCCTACGC

TRPV1-Reverse: CGTAGCAACACCAGCCCAA

Product length = 415 bp

Actin-Forward: CATCCGTAAAGACCTCTATGCCAAC

Actin-Reverse: ATGGAGCCACCGATCCACA

Product length = 171 bp

### Statistical analysis

All data were expressed as the mean ± SEM. All statistical analyses were carried out by using SPSS 16.0. Comparison between two groups was conducted by unpaired Student's *t*-test, comparison among more than two groups was made by One-Way analysis of variance (ANOVA) followed by the least significant difference (LSD) test, and the development of seizure score was analyzed by using a repeated measure ANOVA test. The differences were considered statistically significant if *p* < 0.05.

## Results

### The effects of systemic administration of agonist and antagonist of TRPV1 on PTZ-induced seizures

**Figure 1:**
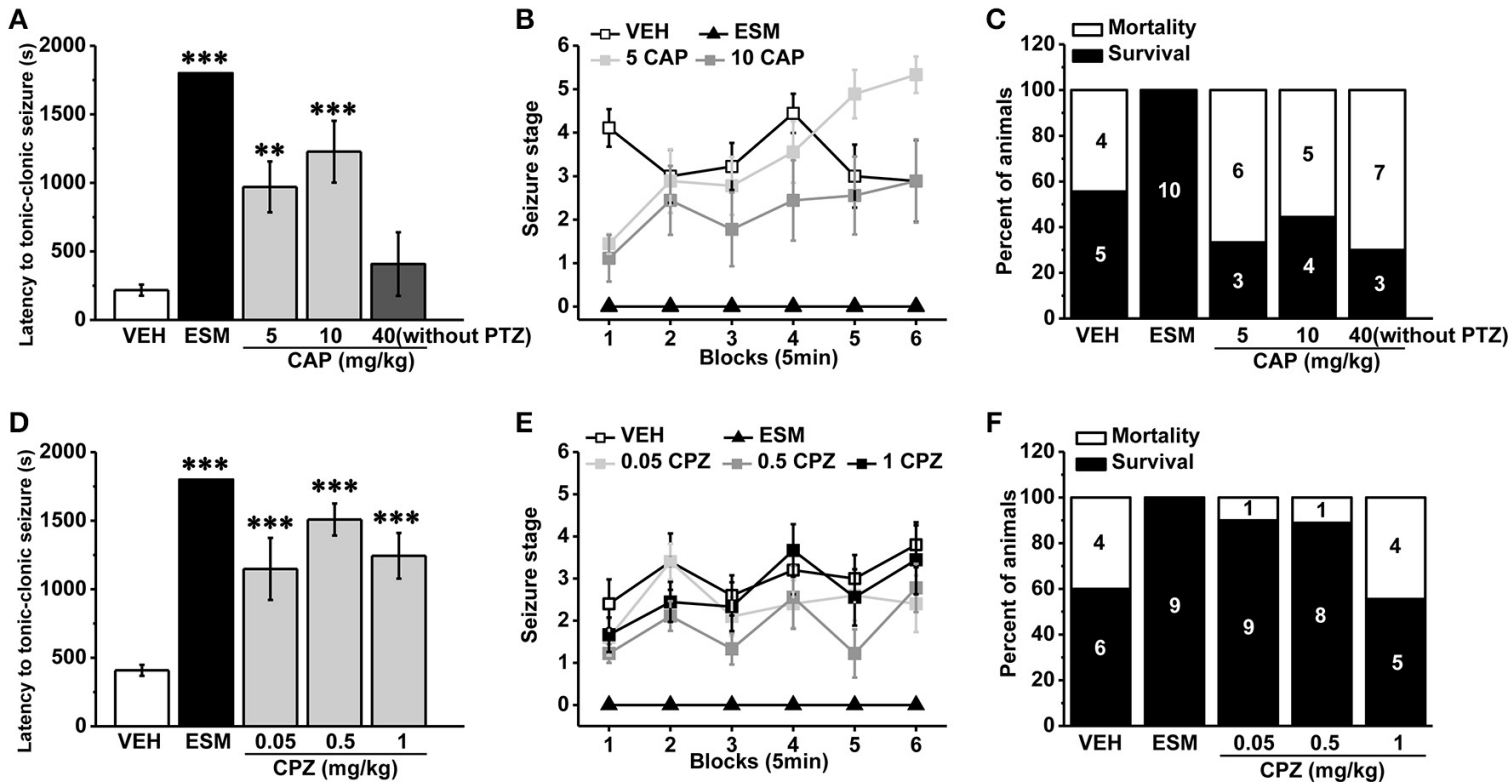
**Systemic administration of TRPV1 agonist CAP increased the severity of seizure while the antagonist CPZ reduced the susceptibility to PTZ-induced seizures. (A)** Injection of CAP (40 mg/kg, i.p.) immediately induced TCS even without PTZ induction. In contrast, the latency to TCS was significantly delayed in 5, 10 mg/kg CAP, and ESM groups compared with the vehicle group. **(B)** Both 5 and 10 mg/kg CAP had the tendency to increase the grades of seizure except ESM compared with the vehicle group. **(C)** 5, 10, and 40 mg/kg CAP increased the mortality except ESM compared with the vehicle mice. **(D)** The latency to TCS was significantly increased in 0.05, 0.5, 1 mg/kg CPZ, and ESM groups compared with the control group. **(E)** 0.05, 0.5, and 1 mg/kg CPZ had no effect on the grades of PTZ-induced seizures except ESM. **(F)** 0.05, 0.5 mg/kg CPZ, and ESM groups showed decreased mortality but not in 1 mg/kg CPZ group compared with the vehicle group. The number in the bars of panel **(C, F)** represents the mice number of death or survival, respectively. All data are expressed as mean ± SEM. ^**^p < 0.01. ^***^p < 0.001.

As a non-competitive antagonist of GABAA receptor, pentylenetetrazol (PTZ) is often used to induce tonic-clonic seizures (TCS) in the animal model of epilepsy (Olsen, 1981; Dhir, 2012). We administrated the agonist and antagonist of TRPV1 respectively 30 min before PTZ (s.c. 60 mg/kg) injection. Ethosuximide (ESM, 625 mg/kg) as a positive control blocked TCS significantly (see Figure 1, latency for ESM: 1800 ± 0 s, *n* = 10, *p* < 0.001; mortality/total number = ESM: 0/10). We found that the specific agonist of TRPV1 capsaicin (CAP, 40 mg/kg) directly led to TCS without PTZ induction, and most animals died (mortality/total number = 7/10) from TCS during the first 5 min after CAP injection (latency: 407.4 ± 232.22 s, *n* = 10). The latency to TCS induced by PTZ was significantly delayed in lower doses of CAP (5 and 10 mg/kg) groups compared with the vehicle group (Figure 1A, vehicle [VEH]: 217.56 ± 40.40 s, *n* = 9; 5 mg/kg CAP [5 CAP]: 970.44 ± 185.77 s, *n* = 9, *p* = 0.006; 10 mg/kg CAP [10 CAP]: 1227.56 ± 225.34 s, *n* = 9, *p* < 0.001), and there is no difference among vehicle, 5, and 10 mg/kg CAP groups in seizure development [Figure 1B, *F*_(2, 24)_ = 1.668, *p* = 0.210]. However, the mortality (Figure 1C, mortality/total number = VEH: 4/9; 5 CAP: 6/9; 10 CAP: 5/9) was increased in both 5 and 10 mg/kg CAP groups. These results showed that activation of TRPV1 with lower dose of CAP increased seizure severity although delayed the latency of PTZ-induced TCS.

TRPV1 antagonist capsazepine (CPZ), a synthetic compound as the structural analog to CAP molecule (Messeguer et al., 2006), is extensively used as a competitive antagonist in pharmacological studies (Maggi et al., 1993; Walpole et al., 1994; Nguyen et al., 2010). The latency to TCS was significantly increased in 0.05, 0.5, and 1 mg/kg CPZ groups compared with the vehicle group (Figure 1D, VEH: 408.4 ± 39.89 s, *n* = 10; ESM: 1800 ± 0 s, *n* = 9, *p* < 0.001; 0.05 mg/kg CPZ [0.05 CPZ]: 1148.50 ± 226.32 s, *n* = 10, *p* < 0.001; 0.5 mg/kg CPZ [0.5 CPZ]: 1508.56 ± 116.25 s, *n* = 9, *p* < 0.001; 1 mg/kg CPZ [1 CPZ]: 1243.78 ± 166.83 s, *n* = 9, *p* < 0.001). Although the development of PTZ-induced seizures was no difference among vehicle, 0.05, 0.5, and 1 mg/kg CPZ groups [Figure 1E, *F*_(3, 34)_ = 2.539, *p* = 0.073], the mortality was reduced in the 0.05 and 0.5 mg/kg CPZ groups (mortality/total number = VEH: 4/10; ESM: 0/9; 0.05 CPZ: 1/10; 0.5 CPZ: 1/9) but not in the 1 mg/kg CPZ group (mortality/total number = 4/9) (Figure 1F). These results showed that systemic administration of the antagonist of TRPV1 reduced seizure severity in a dose-dependent manner.

### Both TRPV1 knockout mice and hippocampal TRPV1 overexpression mice exhibited reduced susceptibility to PTZ-induced seizures

**Figure 2:**
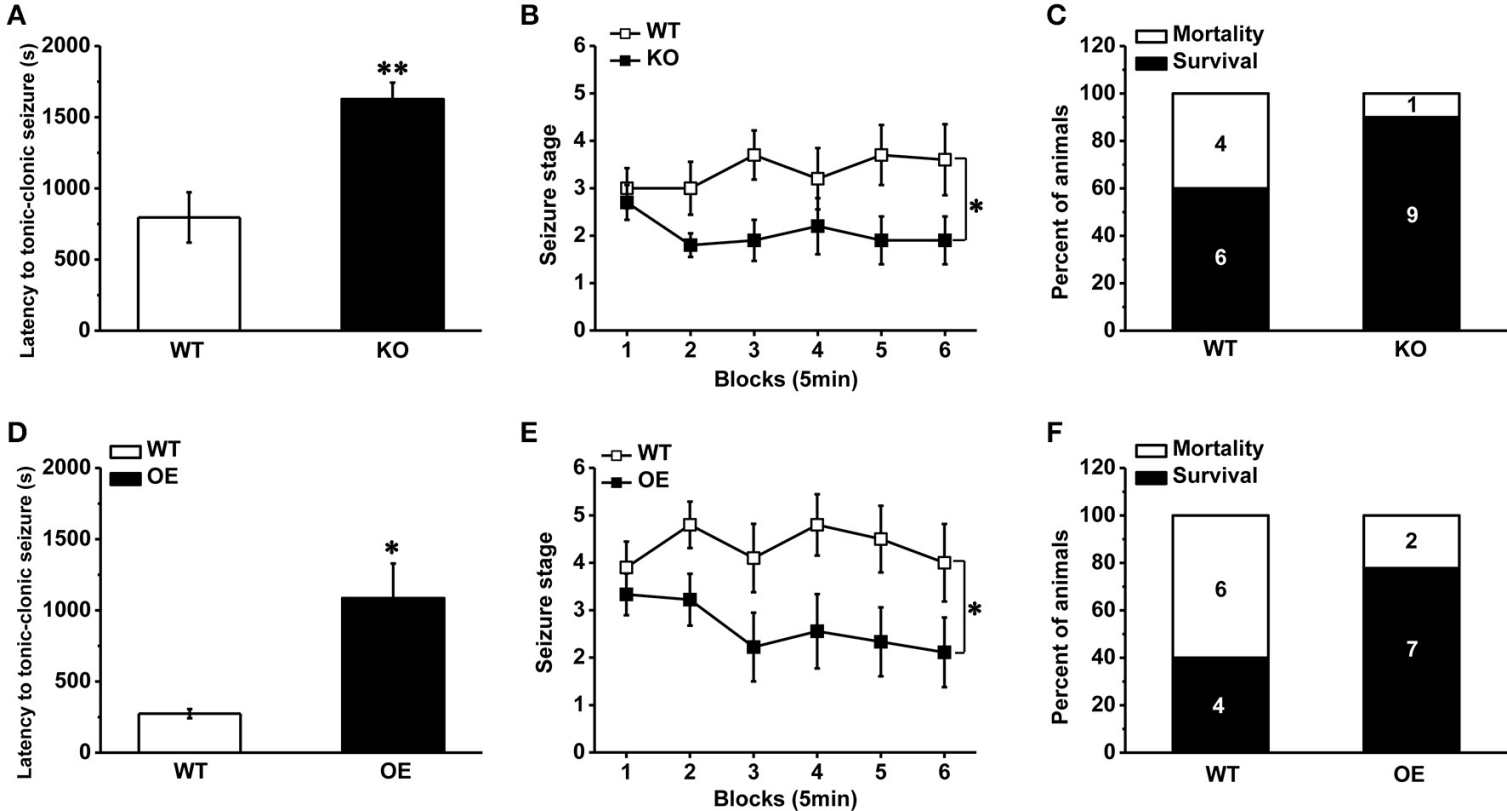
**Decreased susceptibility to PTZ-induced seizures in TRPV1 knockout mice and hippocampal TRPV1 overexpression mice. (A)** The latency to TCS in the TRPV1 knockouts was delayed compared with WT mice. **(B)** The development of seizure was reduced in TRPV1 knockout mice than WT mice. **(C)** The TRPV1 knockout mice also showed decreased mortality. **(D)** Longer latency to TCS of hippocampal TRPV1 overexpression (OE) mice than WT mice. **(E)** Reduced seizure development of hippocampal TRPV1 OE mice compared with the WT group. **(F)** Lower mortality in hippocampal TRPV1 OE mice. All data are expressed as mean ± SEM. ^*^p < 0.05. ^**^p < 0.01.

As a competitive antagonist of TRPV1, CPZ may block other voltage-activated calcium channels (Docherty et al., 1997). In order to avoid the side interference factor of CPZ, we used a transgenic mouse with TRPV1 knockout (Caterina et al., 2000). As described in previous report (Caterina et al., 2000), the TRPV1 gene was disrupted by deleting an exon encoding part of the fifth and all of the sixth putative transmembrane domains of the channel, together with the intervening pore-loop region in the TRPV1 knockout mice. The mice lacking TRPV1 displayed reduced susceptibility to PTZ-induced seizures. The latency to TCS was longer compared with the wild type (WT) mice (Figure 2A, WT: 795.80 ± 176.70 s, *n* = 10; knockout [KO]: 1626.8 ± 116.18 s *n* = 10; *p* = 0.001). And the development of PTZ-induced seizures [Figure 2B, *F*_(1, 18)_ = 5.599, *p* = 0.029] and mortality (Figure 2C, mortality/total number = WT: 4/10; KO: 1/10) were both significantly decreased in TRPV1 knockout mice. Consistent with the results of systemic TRPV1 antagonist CPZ, these results suggested that the decrease of TRPV1 function systemically reduced the susceptibility to PTZ-induced seizures. However, opposite from results of systemic agonist capsaicin, hippocampal TRPV1 overexpression mice exhibited reduced susceptibility to PTZ-induced seizures.

TRPV1 is expressed in the hippocampus, which plays a central role in the generation and propagation of seizure (Wieshmann et al., 1999). However, the role of TRPV1 in the hippocampus in epileptogenesis *in vivo* has not yet been attempted. We generated a transgenic mouse line by crossing ROSA-TRPV1 mice to CA1-Cre mice (as described previously in methods), which overexpressed TRPV1 specifically in the hippocampus. Our results showed that the hippocampal TRPV1 overexpression (OE) mice exhibited reduced susceptibility to seizure. Longer latency to TCS (Figure 2D, WT: 274.40 ± 32.04 s, *n* = 10; OE: 1086.30 ± 242.55 s *n* = 9; *p* = 0.01) and decreased seizure development [Figure 2E, *F*_(1, 17)_ = 6.109, *p* = 0.024] were observed in hippocampal OE mice compared with the WT mice, and the mortality (Figure 2F, mortality/total number = WT: 6/10; OE: 2/9) induced by seizure was decreased in hippocampal OE mice. These findings showed that increase of hippocampal TRPV1 would protect animals away from PTZ-induced seizures, which also suggested that TRPV1 in the hippocampus exerted an important role in epilepsy.

### Hippocampal administration of TRPV1 agonist and antagonist reduced the susceptibilities to PTZ-induced seizures in a dose-dependent manner

**Figure 3:**
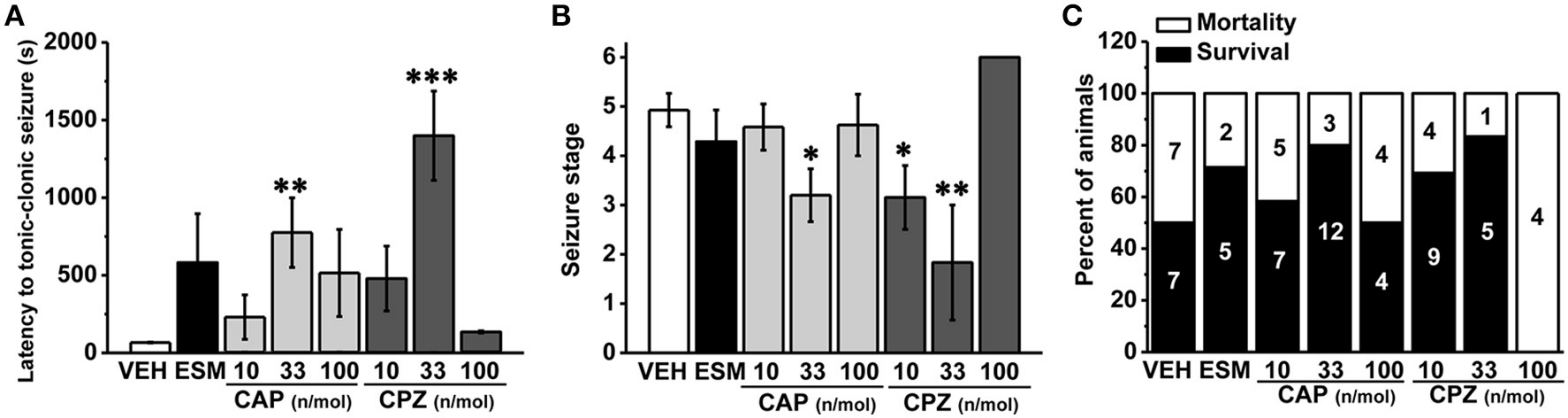
**The susceptibility to PTZ-induced seizures was reduced after intra-hippocampal administration of TRPV1 agonist and antagonist in a dose-dependent manner. (A)** The latency to TCS was significantly delayed in hippocampal administration of 33 nmol/μL/side CAP, 33 nmol/μL/side CPZ groups compared with the vehicle group. The 10, 100 nmol/μL/side CAP; 10, 100 nmol/μL/side CPZ, and ESM had no effect on the latency to TCS. **(B)** Similarly, the maximum grades of seizure were significantly reduced by 33 nmol/μL/side CAP, and 10, 33 nmol/μL/side CPZ except 10, 100 nmol/μL/side CAP, 100 nmol/μL/side CPZ, and ESM compared with the vehicle group. **(C)** Decreased mortality in 33 nmol/μL/side CAP and 10, 33 nmol/μL/side CPZ groups. However, the mortality were not changed by 10, 100 nmol/μL/side CAP, 100 nmol/μL/side CPZ, and ESM administration. All data are expressed as mean ± SEM. ^*^p < 0.05. ^**^p < 0.01. ^***^p < 0.001.

To further investigate the role of hippocampal TRPV1 in the epilepsy, we administrated TRPV1 agonist CAP and antagonist CPZ directly into the hippocampus area. Here we found that the latency to TCS was significantly delayed with intra-hippocampal injection of 33 nmol/μL/side CAP compared with the vehicle group (Figure 3A, VEH: 56.36 ± 1.77 s, *n* = 14; 10 nmol/μL/side CAP [10 CAP]: 230.58 ± 142.98 s, *n* = 12, *p* = 0.505; 33 nmol/μL/side CAP [33 CAP]: 775.20 ± 223.78 s, *n* = 15, *p* = 0.005; 100 nmol/μL/side CAP [100 CAP]: 515.12 ± 280.62 s, *n* = 8; *p* = 0.122). The maximum seizure score (Figure 3B, VEH: 4.93 ± 0.34; 10 CAP: 4.58 ± 0.47, *p* = 0.645; 33 CAP: 3.2 ± 0.54, *p* = 0.017; 100 CAP: 4.63 ± 0.63, *p* = 0.719) and the mortality (Figure 3C, mortality/total number = VEH: 7/14; 10 CAP: 5/12; 33 CAP: 3/15; 100 CAP: 4/8) were reduced after hippocampal administration of 33 nmol/μL/side CAP. However, the behavioral performances of seizure were not changed by CAP at the doses of 10 and 100 nmol/μL/side. On the other hand, the latency to TCS was significantly delayed with TRPV1 antagonist CPZ at the dose of 33 nmol/μL/side compared with vehicle group (Figure 3A, 10 nmol/μL/side CPZ [10 CPZ]: 479.15 ± 208.99 s, *n* = 13, *p* = 0.102; 33 nmol/μL/side CPZ [33 CPZ]: 1399.20 ± 287.78 s, *n* = 6, *p* < 0.001; 100 nmol/μL/side CPZ [100 CPZ]: 135.25 ± 6.61 s, *n* = 4, *p* = 0.834). The maximum seizure score (Figure 3B, 10 CPZ: 3.15 ± 0.65, *p* = 0.018; 33 CPZ: 1.83 ± 1.17, *p* = 0.001; 100 CPZ: 6 ± 0, *p* = 0.323) and the mortality induced by seizure (Figure 3C, mortality/total number = 10 CPZ: 4/13; 33 CPZ: 1/6; 100 CPZ: 4/4) were both decreased in the 10 and 33 nmol/μL/side CPZ groups, not in 100 nmol/μL/side CPZ group. Hippocampal administration of the therapeutic drug ESM had the tendency to increase the latency to TCS (latency: 581.71 ± 314.73 s, *n* = 7, *p* = 0.091), but could not alleviate the seizure development (seizure score: 4.29 ± 0.64, *p* = 0.467) and mortality (mortality/total number = 2/7). It is possible that hippocampal administration of ethosuximide (ESM), the positive control, lacked positive effects on PTZ-induced seizures may be due to the unclear action mechanism of ESM in different brain areas. It was concluded that both agonist and antagonist of hippocampal TRPV1 were efficacious in anti-seizure effects in a dose-dependent manner, which provided further evidence that hippocampal TRPV1 participated in epileptogenesis.

### The confirmation of genetic system and PTZ induction led to the enhancement of hippocampal TRPV1 expression

**Figure 4:**
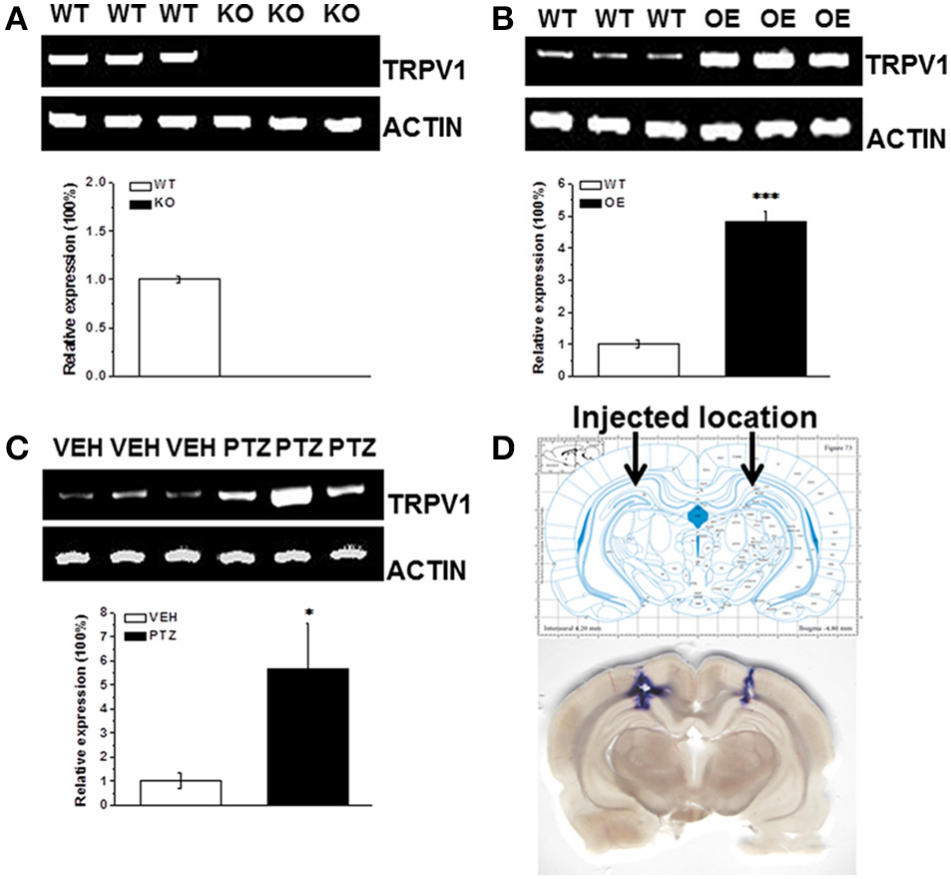
**The RT-PCR analysis of TRPV1 expression in hippocampus. (A)** The TRPV1 expression was lost in the hippocampus of TRPV1 knockout mice compared with the wild type mice. **(B)** The RT-PCR revealed increased expression of hippocampal TRPV1 in TRPV1 overexpression mice compared with the wild type mice. **(C)** The hippocampal TRPV1 expression was significantly increased in PTZ group compared with the vehicle group. **(D)** Schematic diagram represented the drug injection location in hippocampus (Top panel). The bottom picture was the representative section displaying the location of drug injection in hippocampus by stained with trypanblue from a typical rat. TRPV1: 415 bp; β-actin: 171 bp. All data are expressed as mean ± SEM. ^*^p < 0.05. ^***^p < 0.001.

We did detect the genotype of each mouse by PCR. And we showed the genotyping of TRPV1 knockout mice and hippocampal TRPV1 OE (Supplementary Figure 1). To further verify our transgenic system, we detected TRPV1 expression in hippocampus by RT-PCR analysis. The TRPV1 expression was lacked in the hippocampus of TRPV1 knockout mice (Figure 4A). Meanwhile, hippocampal TRPV1 expression was significantly enhanced in the hippocampal TRPV1 OE mice compared with the wild type mice (Figure 4B, WT: 1 ± 0.13, *n* = 3; OE: 4.84 ± 0.31, *n* = 3, *p* < 0.001). These results confirmed that the genetic system here worked well.

As mentioned above that alteration of hippocampal TRPV1 expression changed the susceptibility to PTZ-induced seizures, we are wondering whether PTZ induction altered the expression of TRPV1 in hippocampus. We further used RT-PCR to test the hippocampal TRPV1 expression after PTZ induction. The TRPV1 expression in hippocampus was significantly increased after PTZ-induction compared with the vehicle treated group (Figure 4C, VEH: 1 ± 0.33, *n* = 3; PTZ: 5.65 ± 1.9, *n* = 3, *p* = 0.038), which is consistent with previous study in patients (Sun et al., 2013).

Moreover, we used microinjection to administer 1 μL/side trypanblue into hippocampus to show the actual location of rat hippocampus, and the location was corresponded to the example picture from “The Rat Brain in Stereotaxic Coordinates” (Figure 4D).

## Discussion

It is concluded that systemic or hippocampus-specific interference of TRPV1 altered the susceptibility of PTZ-induced seizures in our present investigation. Systemic administration of TRPV1 antagonist CPZ reduced the susceptibility of PTZ-induced seizures in a dose-dependent manner while the agonist increased the seizure severity although delayed the latency to TCS. Furthermore, TRPV1 knockout mice exhibited decreased susceptibility of PTZ-induced seizures. Interestingly, the reduced susceptibility of PTZ-induced seizures was also observed in hippocampal OE mice. In addition, hippocampal administration of TRPV1 agonist or antagonist reduced the susceptibility to PTZ-induced seizures. Taken together, our findings support that systemic administration of TRPV1 antagonist may be a novel therapeutic target for epilepsy, and hippocampal TRPV1 plays an important role in epileptogenesis.

Our present results showed that systemic administration of TRPV1 agonist CAP at high dose (40 mg/kg) directly induced TCS without PTZ induction, and most of the animals died at the first 5 min after CAP injection. It is supposed that excessive activation of TRPV1 may lead to cytotoxicity and neuronal death process (Shirakawa et al., 2008; Davies et al., 2010), thus high dose CAP may directly produce the onset of seizure. In addition, we showed that the lower doses of CAP increased the severity of PTZ-induced seizures, which was similar with previous reports (Manna and Umathe, 2012; Gonzalez-Reyes et al., 2013). However, opposite from results of **s**ystemic CAP, direct administration of TRPV1 agonist CAP into hippocampus decreased seizure severity, which may be resulted from the desensitization of hippocampal TRPV1 by CAP. Iannotti et al. found that CAP reduced both epileptiform burst amplitude and duration in hippocampal brain slices exposed to Mg^2+^-free solution by TRPV1 de-phosphorylation and desensitization (Iannotti et al., 2014). Furthermore, various types of TRPV1 expressing cell showed different desensitization patterns by CAP (Czikora et al., 2013), the different effects of TRPV1 on different brain regions were integrated in the systemic administration, which may explain for the increased seizure severity in systemic CAP administration experiment. Thus it is understandable the inconsistent results from systemic and hippocampal injection of TRPV1 agonist. The delayed latency but increased severity of PTZ-induced TCS in lower doses of systemic CAP experiment may be caused by the desensitization upon activation (Figure 1). The effects of TRPV1 agonist on epilepsy will be studied further in consideration of TRPV1 desensitization and its different effects on various brain areas in future research.

It has been reported that TRPV1 is expressed at relatively high level in the human hippocampus (Mezey et al., 2000; Sun et al., 2013), a focus of seizure generation and propagation (Wieshmann et al., 1999). The expression of TRPV1 protein was significantly increased in the hippocampus and cortex of subjects with epilepsy (Bhaskaran and Smith, 2010; Sun et al., 2013). Similarly, our RT-PCR results revealed that PTZ-induced seizure increased hippocampal TRPV1 expression (Figure 4C). Hippocampal TRPV1 OE mice showed increased quantity of TRPV1 expression in the hippocampus (Figure 4B), which may increase neuronal excitability via the enhanced influx of calcium. However, we did not observe any sign of spontaneous seizure in these mice, which indicated that homeostatic regulation of intrinsic excitability has been established in hippocampal TRPV1 OE mice. To be notified that opposite from results of systemic agonist CAP, hippocampal TRPV1 OE mice exhibited reduced susceptibility to PTZ-induced seizures. We speculated that due to the ceiling effect of overexpressed hippocampal TRPV1 in these mice, PTZ induction could not further increased TRPV1 expression and excitability in hippocampus, which may explain for the decreased susceptibility to PTZ-induced seizure in hippocampal TRPV1 OE mice. Furthermore, it was also supposed that TRPV1 may predominantly overexpress on GABA-interneurons thus reduced the excitability. Therefore, the cellular specific distribution of TRPV1 expression needs to be clarified in future research.

Several findings indicated that TRPV1 antagonist reduced the propagation of epileptiform activity (Gonzalez-Reyes et al., 2013) and prevented the PTZ-induced seizures (Manna and Umathe, 2012). In our investigation, systemic administration of TRPV1 antagonist CPZ significantly delayed the latency to TCS and decreased the mortality induced by seizure (Figures 1D–F), which was consistent with previous study (Manna and Umathe, 2012). Furthermore, we introduced TRPV1 knockout mice (Caterina et al., 2000) to further determine its function in epilepsy. Kong et al. previously reported that lack of TRPV1 attenuated the severity of PTZ -induced seizures (Kong et al., 2014). Consistent with this report, we also observed that the mice lack of TRPV1 displayed longer latency to TCS, reduced seizure development and mortality (Figures 2A–C). Both results from CPZ administration and knockout mice showed that suppression of TRPV1 function prevented PTZ-induced seizures. It was unfortunate that we did not obtain a conditional hippocampal TRPV1 knockout mouse line. However, we have injected TRPV1 antagonist CPZ into hippocampus, and found that suppression of hippocampal TRPV1 delayed the latency to TCS, reduced the development of seizure and mortality in a nice dose-dependent manner (Figures 3A–C). Here we firstly reported that suppression of hippocampal TRPV1 reduced the susceptibility to PTZ-induced seizures. Combined with the results from systemic administration of TRPV1 antagonist and TRPV1 knockout mice, we demonstrated that the suppression of function of TRPV1 exerted an anti-seizure property.

## Conclusions

In this investigation, we found that suppression of TRPV1 by systemic or hippocampal administration of antagonist or lack of TRPV1 reduced the susceptibility to PTZ-induced seizures. Furthermore, hippocampal administration of TRPV1 agonist and hippocampal TRPV1 OE mice exhibited decreased susceptibility of seizure, although the systemic administration of TRPV1 agonist led to increased seizure severity. Therefore, it remains to be further studied the underlying mechanism of hippocampal TRPV1 function in epilepsy. In summary, our results indicate that systemic administration of TRPV1 antagonist may be a new potential therapeutic target for epilepsy treatment, and hippocampal TRPV1 exerts important role in epilepsy.

### Conflict of interest statement

The authors declare that the research was conducted in the absence of any commercial or financial relationships that could be construed as a potential conflict of interest.

## Supplementary material

Supplementary Figure 1 **Genotyping of TRPV1 knockout mice and hippocampal TRPV1 overexpression mice. (A)** The genotype of TRPV1 knockout mice. Mutant = 600 bp; Heterozygote = 600 bp and 984 bp; Wild type = 984 bp. **(B)** The hippocampal TRPV1 overexpression mice were crossed by CA1-Cre (top panel) with ROSA26-TRPV1 (bottom panel) mice, both of which were genotyped by specific primers respectively. Positive Cre = 300–400 bp; Negative Cre = no band. ROSA-TRPV1: Mutant = 600 bp; Heterozygote = 600 bp and 482 bp; Wild type = 482 bp. M = Mutant; W = Wild type; H = Heterozygote; P = Positive Cre; N = Negative Cre.
