# Supplementary figures and images for: Interference of TRPV1 function altered the susceptibility of PTZ-induced seizures
Source: Front Cell Neurosci. 2015 Feb 10;9:20. doi: 10.3389/fncel.2015.00020 (PMC4322730; doi:10.3389/fncel.2015.00020)

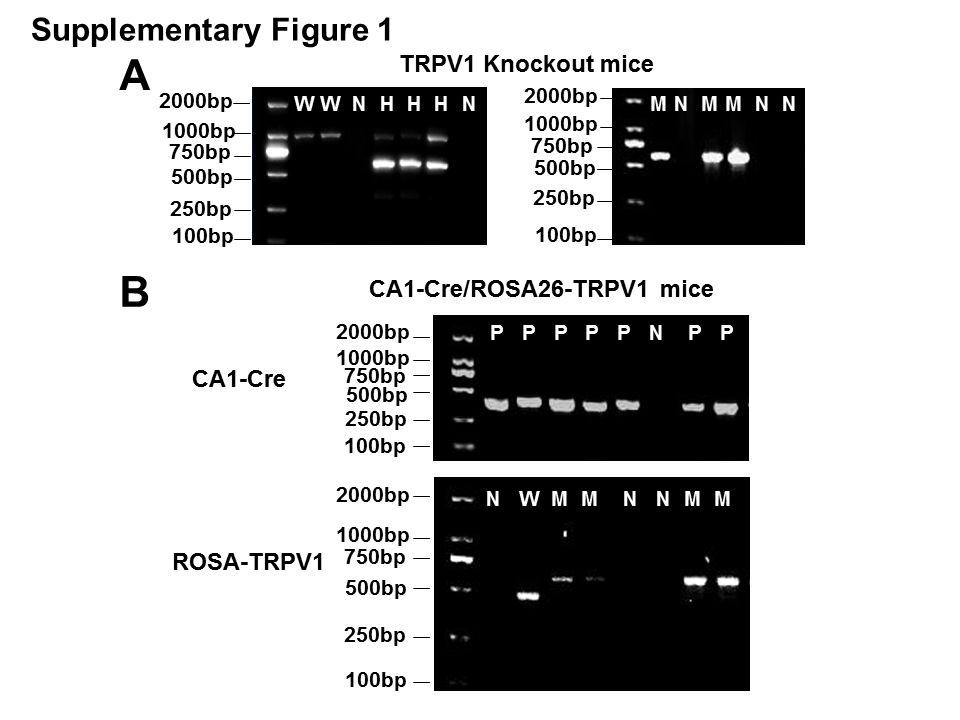

Supplement: Supplementary Figure 1 — Genotyping of TRPV1 knockout mice and hippocampal TRPV1 overexpression mice. (A) The genotype of TRPV1 knockout mice. Mutant = 600 bp; Heterozygote = 600 bp and 984 bp; Wild type = 984 bp. (B) The hippocampal TRPV1 overexpression mice were crossed by CA1-Cre (top panel) with ROSA26-TRPV1 (bottom panel) mice, both of which were genotyped by specific primers respectively. Positive Cre = 300–400 bp; Negative Cre = no band. ROSA-TRPV1: Mutant = 600 bp; Heterozygote = 600 bp and 482 bp; Wild type = 482 bp. M = Mutant; W = Wild type; H = Heterozygote; P = Positive Cre; N = Negative Cre. [file Image1.TIF]
